# Supplementary material for: The Epidemiology of Skin Cancer and Public Health Strategies for Its Prevention in Southern Africa
Source: Int J Environ Res Public Health. 2020 Feb 6;17(3):1017. doi: 10.3390/ijerph17031017 (PMC7037230; doi:10.3390/ijerph17031017)
Supplement: Supplementary file 1 [file ijerph-17-01017-s001.pdf]

Supplementary Table S1. Absence (x) or presence (✓) of evidence of online skin cancer prevention campaigns (any focus – could be in relation to albinism) in English by country in Africa. Websites valid as at the 17 December 2019.

Country Name	Absence (x) or presence (✓) of an online skin cancer prevention campaign or skin cancer information	Description of online skin cancer prevention campaign and information	Websites of skin cancer information and / or skin cancer prevention campaigns
Botswana	✓	Botswana has a Cancer Association and information about the association is given on the Global Cancer Control website. The Botswana Cancer Association website address is not functional. 'Botsogo' is the Botswana Oncology Global Outreach that aims to improve access to cancer care in Botswana. There is no specific mention of skin cancer on these websites.	https://www.uicc.org/membership/cancer-association-botswana<br><br>http://www.botsogo.org/<br><br>http://www.cancer.org.bw
Swaziland	x	The Swaziland National Cancer Registry has a presence on the African Cancer Registry Network but there was no Internet presence of a Cancer Association promoting skin cancer prevention for Eswatini.	https://afcrn.org/index.php/membership/membership-list/146-swazilandncr
Lesotho	✓	There was no Internet presence of a Cancer Association promoting skin cancer prevention for Lesotho. However, at least two websites were identified that promoted skin cancer protection among people living with albinism.	https://african-albinos.org/ons-werk/albinisme-in-lesotho/<br><br>https://actiononalbinism.org/page/v1p6dpzt34
Namibia	✓	The Cancer Association of Namibia has provided sun protection information to the country via a newsletter entitled, 'Acacia' in 2019.	https://www.can.org.na/<br><br>https://www.can.org.na/wp-content/uploads/2019/08/Acacia-News-2019-vol-2-1.pdf

		Support in Namibia of Albinism Sufferers Requiring Assistance is entity that provides sunscreen, clothing and sunglasses to people with albinism.	https://sinasra.com/
South Africa	✓	The Cancer Association of South Africa has a dedicated webpage for skin cancer prevention and sun protection.<br><br>The Cancer Association of South Africa also highlights skin cancer risk among people with albinism.<br><br>The Skin Cancer Foundation of South Africa and the Dermatological Society of South Africa also provide skin cancer prevention information.<br><br>The Albinism Society for South Africa's website is presently not active (22 November 2019), however, anecdotal evidence suggests that the society advocates for sun protection support for people with albinism in the country.	https://www.cansa.org.za/sunsmart-radio-spots-featuring-uv-rays-sunny-brand/<br><br>https://www.cansa.org.za/albinism-increases-skin-cancer-risk-in-south-africa/<br><br>https://skincancerfoundation.co.za/<br><br>https://derma.co.za/<br><br>http://www.albinism.org.za/
